# MPF Regulates Oocyte and Embryo Development During Parthenogenesis Induction in Silkworm, *Bombyx mori*

**DOI:** 10.3390/insects16040361

**Published:** 2025-03-31

**Authors:** Chenkai Ma, Fang Xu, Chengjie Hu, Chunguang Cui, Xin Du, Jine Chen, Linbao Zhu, Shaofang Yu, Xingjian He, Wei Yu, Yongqiang Wang, Xia Xu

**Affiliations:** 1 College of Life Sciences and Medicine, Zhejiang Sci-Tech University, Hangzhou 310018, Chinayuwei@zstu.edu.cn (W.Y.); 2 Institute of Sericulture and Tea, Zhejiang Academy of Agricultural Sciences, Hangzhou 310021, China

**Keywords:** MPF, enzyme activity, embryo development, parthenogenesis, *Bombyx mori*

## Abstract

In most species, oocytes are arrested at the prophase or metaphase of meiosis I and require sperm-derived or external stimuli to resume meiosis. Maturation-promoting factor (MPF) is an oocyte maturation factor composing the catalytic subunit Cdc2 and the regulatory subunit CycB that can restart stalled meiosis. In this study, we demonstrated that MPF activity affected parthenogenesis induction in the model lepidopteran insect *Bombyx mori* using activator and inhibitor interference. We found that the upregulation of MPF activity significantly increased the parthenogenesis induction rate, whereas downregulation significantly reduced it. Furthermore, the inhibition of MPF activity also led to a delay in embryonic development. Given its evolutionary conservation, MPF emerges as a potential universal target for manipulating reproductive outcomes, offering broad applications in genetics and selective breeding.

## Simple Summary

The unfertilized eggs of silkworm treated in a warm bath (46 °C, 18 min) can induce parthenogenesis, resulting in progeny that are exclusively female and genetically identical. This process involves dynamic changes during oocyte division, which are directly related to the cell cycle. Maturation-promoting factor (MPF) played a crucial role in regulating oocyte meiosis, ensuring normal fertilization and subsequent embryonic development. MPF activity was proportional to the silkworm parthenogenesis induction rate, and the induction rate varied regularly with the change in the activity. Additionally, it was observed that embryonic development was delayed after parthenogenesis induction by the inhibitor interference. These insights hold significant promise for advancing genetic and breeding research.

## 1. Introduction

The silkworm, *Bombyx mori*, serves as a pivotal lepidopteran model organism in genetic and genomic research [1,2,3,4]. Under natural conditions, silkworms typically reproduce sexually, although unfertilized eggs may occasionally develop into new individuals through parthenogenesis [4]. However, the natural incidence of parthenogenesis in silkworm is extremely low, at only 0.003% [5,6]. Unfertilized silkworm eggs can be artificially induced to develop using physical or chemical methods. One of the most widely used techniques involves subjecting the eggs to a warm bath (46 °C, 18 min), which induces ameiotic parthenogenesis (AMP) in silkworm [4]. AMP refers to the absence of allelic recombination during oocyte meiosis, resulting in offspring that are genetically identical to the maternal parent [7]. Although the cytological mechanism of parthenogenesis is well documented, the molecular mechanism of AMP induced by warm bath treatment in silkworm remains to be fully understood.

Oocyte development is arrested at the metaphase of the first meiosis and requires stimulation by sperm or other external factors to resume meiosis in silkworm [8,9]. Interestingly, warm bath induction did not restart the stalled oocyte meiosis in silkworm. Central to cell cycle regulation is maturation-promoting factor (MPF), a heterodimeric complex comprising the catalytic subunit cell division cycle 2 (Cdc2) and the regulatory subunit cyclin B (CycB) [10,11]. Cdc2 is central to the regulation of the G2/M phase transition in the cell cycle [12,13]. It exhibits serine/threonine kinase activity and catalyzes the phosphorylation of various target proteins, thereby ensuring the orderly progression of the cell cycle [14,15,16]. Cdc2 activity is tightly regulated by CycB, whose expression varies during the cell cycle to further regulate MPF activity [17,18].

MPF is indispensable for meiotic resumption during oocyte maturation [19,20]. In *Xenopus laevis*, MPF accumulated as an inactive complex during oocyte development, and only when CycB reached a certain amount did it switch to an activated state, thus initiating the G2-to-M phase transition and initiating cell meiosis [21,22]. Similarly, in *Sus scrofa*, Cdc2 deficiency led to meiotic failure in small oocytes, and the induction of Cdc2 expression enables the completion of meiotic maturation [23,24]. In *Mus musculus*, phosphatase was decisive for MPF to restore oocyte meiosis [25]. During sexual reproduction, MPF ensures proper oocyte meiosis, fertilization, and embryonic development [26,27]. In mammals, the disruption of Cdc2 activity interferes with MPF function and affected parthenogenesis induction [28]. However, the role of MPF in parthenogenesis induction in insects, such as the silkworm, remains poorly understood and warrants further investigation.

In this study, we investigated the function of MPF in warm-bath-induced parthenogenesis in *B. mori*, a model lepidopteran insect. We found that the mRNA expression levels of *Cdc2* and *CycB* were significantly higher in the parthenogenetic line (Wu14) and corresponding amphigenetic line (54A) following the warm bath treatment of silkworm eggs. MPF activity was found to be directly proportional to the parthenogenesis induction rate, with the induction rate exhibiting a consistent pattern in response to changes in MPF activity. Additionally, embryonic development was delayed after parthenogenesis induction when MPF activity was inhibited, whereas early development was not triggered by MPF activation. Perturbations in MPF activity disrupt oocyte cell cycle regulation, leading to parthenogenesis failure. Phylogenetic analysis further revealed that MPF was highly conserved evolutionarily. These findings not only enhance our understanding of the mechanisms underlying parthenogenesis in silkworms but also provide insights that can be applied to population regulation in other species.

## 2. Materials and Methods

### 2.1. Silkworm and Cell Lines

The parthenogenetic line (Wu14) and corresponding amphigenetic line (54A) were bred and provided by Zhejiang Academy of Agricultural Sciences. Wu14 was derived from 54A through warm bath (46 °C, 18 min) induction [6]. Both lines had been bred for over 30 generations, ensuring stable genetic and phenotypic characteristics. The parthenogenetic line Wu14 was derived from the amphigenetic line 54A through warm bath breeding. The amphigenetic line 54A typically reproduces through the fusion of sperm and egg after mating between males and females. We induced unfertilized eggs of 54A using warm bath treatment and reared the individuals that exhibited pigmentation and successful hatching. After these individuals developed into adults, we continued to apply warm bath treatment to their unfertilized eggs. Through over 30 consecutive generations of selective induction, both the pigmentation rate and hatching rate of Wu14 stabilized at over 80%. In other words, the first generation of Wu14 originated from 54A, and subsequent generations were developed through warm bath induction and selective breeding. Silkworm eggs were incubated at 25 °C and 80% relative humidity (RH) for 10 days. The instar larvae were reared on fresh mulberry leaves at 25 °C and 70% RH. To break diapause and sustain continuous rearing, the eggs were treated with hydrochloric acid. The eggs were immersed in 15% hydrochloric acid at 46 °C for 5 min and then placed at 25 °C and 80% RH until hatching.

### 2.2. Artificially Induced Parthenogenesis

Next, 12 h after emergence, female moths were dissected the abdomen, and unfertilized eggs were collected for parthenogenesis induction [4]. The unfertilized eggs were soaked in a warm bath at 46 °C for 18 min, followed by rapid cooling in a room temperature water bath at 25 °C for 3 min. After drying, the eggs were placed in an incubator at 16 °C and 80% RH for 3 days. At this stage, the eggs corresponded developmentally to the post-oviposition stage of sexual reproduction. To break diapause, the eggs were immersed in 15% hydrochloric acid at 46 °C for 5 min and then left at 25 °C and 80% RH until hatching. Following parthenogenesis activation, embryonic development was marked by a change in egg color from yellow to dark due to serous pigmentation on the third day [6]. However, not all pigmentated eggs hatched successfully. The pigmentation and hatching rates of eggs in each moth were calculated (*n* = 42). Pigmentation rate (%) = (number of pigmentated eggs/total number of eggs) × 100%. Hatching rate (%) = (number of hatched eggs/total number of eggs) × 100%.

### 2.3. Protein Structure and Phylogenetics Analysis

The protein structure was predicted using the online software ProtScale for hydrophobicity (https://web.expasy.org/protscale/ (accessed on 20 January 2025)), Uniprot for sequence annotation (https://www.uniprot.org/ (accessed on 20 January 2025)), Sopma for secondary structure (https://npsa-prabi.ibcp.fr/cgi-bin/npsa_automat.pl?page=/NPSA/npsa_sopma.html (accessed on 20 January 2025)), and Swiss-model for 3D modeling (https://swissmodel.expasy.org/ (accessed on 20 January 2025)) [29]. Evolutionary relationships were inferred using the neighbor-joining (NJ) method. Tests were performed by Bootstrap (1000 replicates). Evolutionary distances were computed using the Poisson correction method. Evolutionary analyses were conducted in MEGA 11 [30].

### 2.4. RNA Isolation, cDNA Synthesis, and qPCR Analysis

Total RNAs were isolated using Trizol^®^ reagent (Invitrogen, Carlsbad, CA, USA). For complementary DNA (cDNA) synthesis, 1 μg of total RNA was reverse-transcribed using the RevertAid™ First Strand cDNA Synthesis Kit (Thermo Fisher Scientific, Waltham, MA, USA). Quantitative real-time PCR (qRT-PCR) analyses were performed using a SYBR Green Realtime PCR Master Mix (Thermo Fisher Scientific, Waltham, MA, USA). The PCR conditions were as follows: initial incubation at 95 °C for 5 min, 35 cycles at 95 °C for 15 s, and 60 °C for 1 min. *Ribosomal protein 49* (*Bmrp49*) was used as an internal control [31]. A relative quantitative method (^ΔΔ^Ct) was used to evaluate quantitative variation. The gene-specific primers used for qRT-PCR are listed in Table 1.

### 2.5. Enzyme Inhibition and Activity Detection

Healthy individuals with consistent pupation times were selected and placed in an incubator at 25 °C and 80% RH. Since Cdc2 is a central component of MPF, we modulated MPF activity by targeting Cdc2 using an activator (HY-129478, MCE, Atlantic City, NJ, USA) and an inhibitor (HY-12529, MCE, Atlantic City, NJ, USA) [32,33]. The activator and inhibitor were separately injected into the pupa using a microsyringe. The needle was injected vertically into the middle of the third abdominal somite. The pupal stage for both the Wu14 and 54A lines was 13 days, and injections were given on the 12th day (one day before emergence). DMSO/corn oil (1:9, *v*/*v*) was used to prepare different concentrations of the activator and inhibitor. Based on the properties of the compounds and the preliminary experimental concentration test, three appropriate concentrations were finally set (10 µM, 20 µM, and 40 µM). The administered injection was consistently 5 µL. Eggs were collected 12 h after female emergence, and enzyme activity was detected using ELISA kit (YJ370816, Yuanju, Shanghai, China). MPF is composed of the catalytic subunit Cdc2 and the regulatory subunit CycB. Cdc2 exhibits kinase activity only when bound to CycB. The kinase activity of MPF depends on the binding of Cdc2 to cyclin B and the phosphorylation-activated state of the critical site (Thr161) on Cdc2 [34]. The ELISA kit employed a sandwich immunoassay to measure MPF activity, specifically targeting the Thr161 phosphorylation site of Cdc2, which served as its molecular switch [19,34]. Antibody capture: Antibodies were captured via immobilization on the microplate surface, where they bound to a specific epitope of Thr161-phosphorylated Cdc2, anchoring to the solid phase. Antibody detection: Antibodies were detected via conjugation with horseradish peroxidase (HRP). A spatially distinct epitope was recognized on the same phosphorylated Cdc2 molecule, forming a stable “antibody–antigen–antibody” sandwich complex. Dual-epitope recognition: The requirement for simultaneous binding by both antibodies ensured the detection of intact MPF complexes (Cdc2-CycB with Thr161 phosphorylation) while excluding partially degraded products or nonspecifically phosphorylated proteins. The substrate TMB was added for color development. TMB was converted to a blue color with the catalysis of HRP and subsequently turned yellow in the presence of acid. The intensity of the yellow color was directly proportional to the MPF concentration in the sample. Absorbance was measured at 450 nm with activity proportional to signal intensity.

### 2.6. Ovariole and Embryo Observation

Female moths 12 h after emergence were dissected, and 8 ovarioles were carefully separated using tweezers in insect phosphate-buffered saline (PBS). Images were captured using a camera (Canon, EOS M3, Tokyo, Japan). We randomly selected and mixed the eggs from 10 female moths and then randomly chose 30 eggs from this mixture for embryo observation. For embryo isolation, 20% KOH solution (100 mL) was heated to boiling in a beaker. Eggs were soaked in KOH solution for 3s and immediately transferred to 60 °C warm water for 3 s. Then, the eggs were placed in a Petri dish with 25 °C water and blown repeatedly with a plastic glue head dropper until the complete embryos were obtained. We transferred embryos onto a glass slide using a plastic glue head dropper, followed by direct observation under a microscope. Images were captured using microscope (TL3000 Ergo, Leica, Wetzlar, Germany).

### 2.7. Statistical Analysis

GraphPad Prism 8.3.0 was used for two-tailed Student’s *t*-test analysis. Three independent replicates were used for each treatment. Means were determined, and error bars show the means ± SEM (standard error of the mean).

## 3. Results

### 3.1. Protein Structures of Cdc2 and CycB

**Figure 1:**
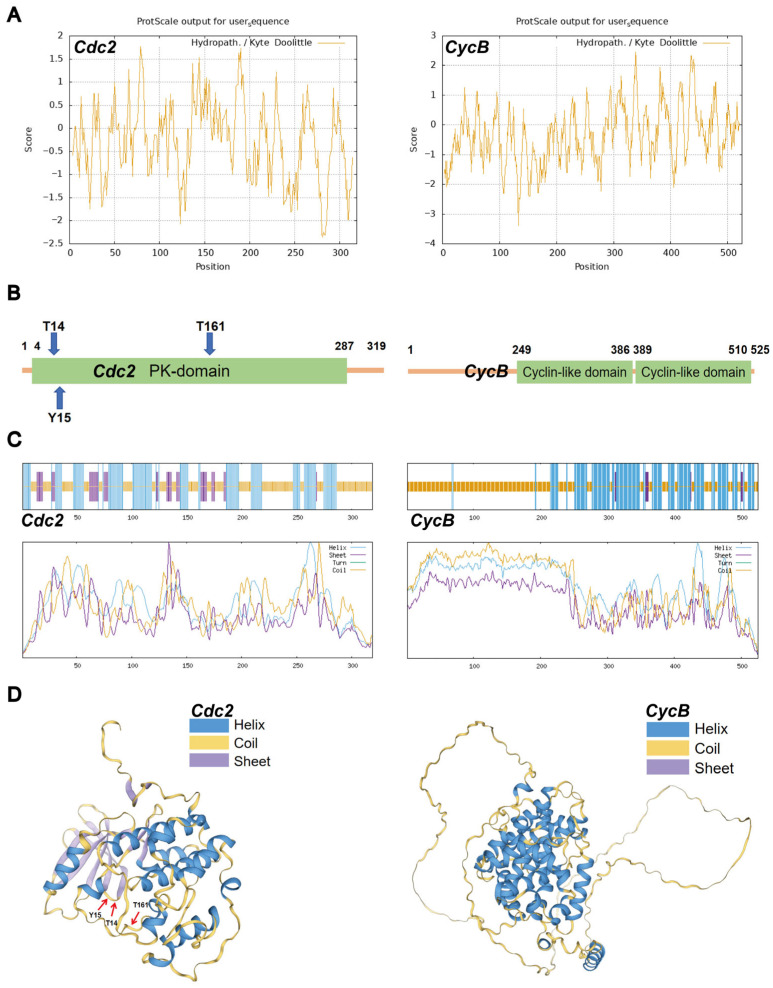
Predicted protein. (**A**) Physicochemical properties of Cdc2 and CycB. The ProtScale was used; a positive score denotes hydrophobic amino acid, and the large value demonstrates strong hydrophobicity; a negative score represents hydrophilic amino acid, and a small value indicates strong hydrophilicity. (**B**) Functional domain of Cdc2 and CycB. Uniprot was used; green is the domain location. (**C**) Secondary structure of Cdc2 and CycB. Sopma was used; blue is the helix; purple is the sheet; green is the turn; yellow is the coil. (**D**) Tertiary structure of Cdc2 and CycB. Swiss-model was used; blue is the helix; purple is the sheet; yellow is the coil.

The catalytic subunit Cdc2 (319 aa, pI 7.69) exhibited strong hydrophilicity with predominant polar amino acid composition (Figure 1A). Its kinase domain (aa 4–287) contained three critical phosphorylation sites: Thr-14 and Tyr-15 (inhibitory) and Thr-161 (activating), all located within random coil regions (Figure 1B). The structural prediction of Cdc2 revealed 17 α-helices (38.87%), 11 β-strands (13.48%), and 17 random coils (47.65%) (Figure 1C,D). And the three phosphorylation sites, Thr-14, Thr-15, and Thr-161, were all located within the random coil regions. The regulatory subunit CycB (525 aa, pI 9.27) contained two cyclin-like domains (aa 249–386 and 389–510) that regulate kinase activity (Figure 1A,B). These domains primarily regulate cyclin-dependent protein serine/threonine kinase activity. The secondary structure of CycB comprised 20 α-helices (40.00%) and 18 random coils (57.90%) (Figure 1C,D). The functional MPF complex is formed through Cdc2-CycB interaction, with Thr161 phosphorylation serving as the molecular switch for cell cycle regulation [35,36].

### 3.2. Phylogenetic Identification of Cdc2 and CycB

**Figure 2:**
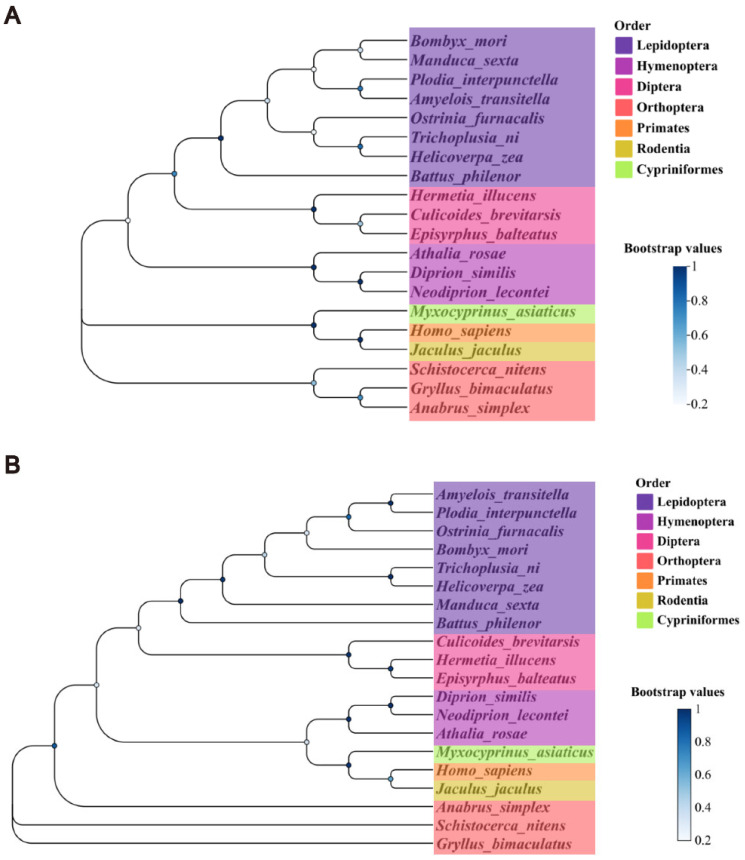
Phylogenetic tree. (**A**) Cdc2. (**B**) CycB. Protein sequences from *B. mori*, *Manduca sexta*, *Trichoplusia ni*, *Ostrinia furnacalis*, *Amyelois transitella*, *Helicoverpa zea*, *Battus philenor*, *Plodia interpunctella*, *Diprion similis*, *Neodiprion lecontei*, *Athalia rosae*, *Hermetia illucens*, *Culicoides brevitarsis*, *Episyrphus balteatus*, *Gryllus bimaculatus*, *Anabrus simplex*, *Schistocerca nitens*, *Homo sapiens*, *Jaculus jaculus*, and *Myxocyprinus asiaticus*.

Homologous sequences of the Cdc2 and CycB proteins were selected from 20 different species to explore evolutionary conservation. These species were as follows: *B. mori*, *Manduca sexta*, *Trichoplusia ni*, *Ostrinia furnacalis*, *Amyelois transitella*, *Helicoverpa zea*, *Battus philenor*, *Plodia interpunctella*, *Diprion similis*, *Neodiprion lecontei*, *Athalia rosae*, *Hermetia illucens*, *Culicoides brevitarsis*, *Episyrphus balteatus*, *Gryllus bimaculatus*, *Anabrus simplex*, *Schistocerca nitens*, *Homo sapiens*, *Jaculus jaculus*, and *Myxocyprinus asiaticus*. Phylogenetic analysis revealed that the Cdc2 and CycB proteins were moderately conserved across these species (Figure 2). Protein kinase and cyclin-like functional domains were highly specific, further indicating that MPF involved in cell cycle was species-specific. These findings indicated that insights into MPF function in *B. mori* are likely applicable to other species. The sequence numbers of the proteins in the corresponding species are listed in Table 2.

### 3.3. Differential mRNA Expression in Parthenogenetic and Amphigenetic Lines

**Figure 3:**
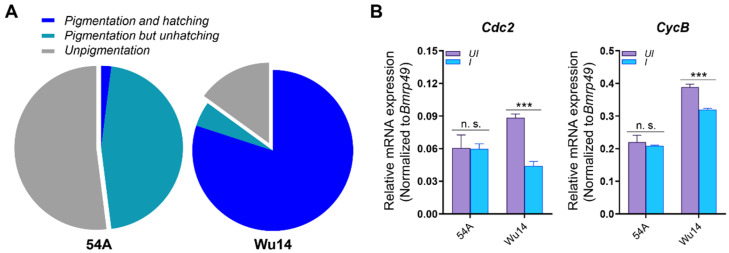
The mRNA expressions in 54A and Wu14. (**A**) Parthenogenesis induction rate. (**B**) mRNA expression. Pigmentation rate is blue and cyan, hatching rate is blue, and unpigmented rate is gray. The mRNA expression level was normalized to *B. mori ribosomal protein 49* (*Bmrp49*), an internal reference. The data shown are means ± S.E.M. (*n* = 3). Asterisks indicate significant differences with a two-tailed *t*-test: *** *p* < 0.001; n. s. *p* > 0.05.

The comparative analysis of Wu14 (parthenogenetic) and 54A (amphigenetic) lines revealed striking reproductive differences. The results revealed that the pigmentation rate of Wu14 exceeded 85%, with a hatching rate of over 80%, while 54A exhibited a pigmentation rate of approximately 50% but a significantly lower hatching rate of less than 2% (Figure 3A). We collected unfertilized eggs of 54A and Wu14, including uninduced eggs (UI) and induced eggs (I) in a warm bath. We detected *Cdk2* mRNA expression levels in both UI and I. Subsequently, we examined the mRNA expression levels of *Cdc2* and *CycB* genes in the eggs. The results showed significant differences in *Cdc2* and *CycB* expression between UI and I in parthenogenetic line Wu14, but no such differences were observed in corresponding amphigenetic line 54A (Figure 3B). These results establish a direct correlation between MPF component expression and parthenogenetic competence.

### 3.4. Activator and Inhibitor Effectively Regulated MPF Activity and Were Not Toxic

**Figure 4:**
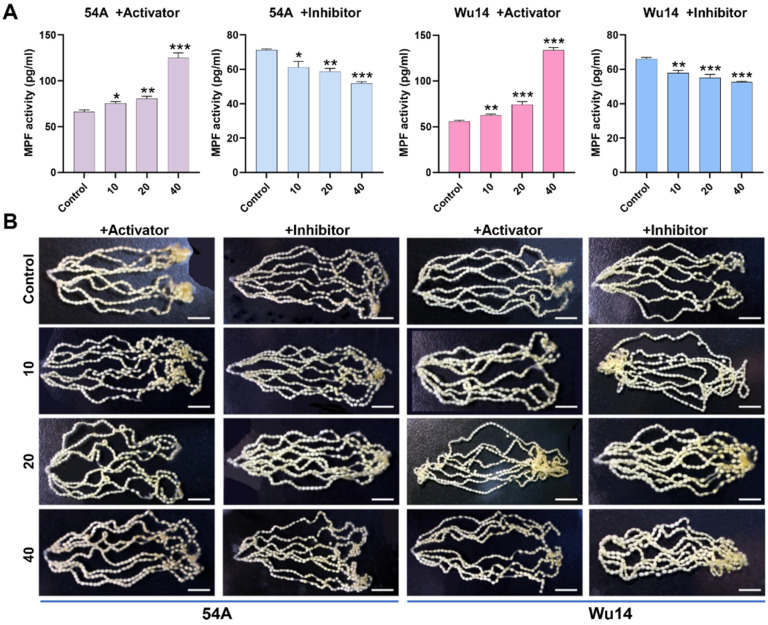
Regulation of MPF activity. (**A**) Enzyme activity. (**B**) Ovariole observation. The unit of concentration was μM. The data shown are means ± S.E.M. (*n* = 3). Asterisks indicate significant differences with a two-tailed *t*-test: * *p* < 0.05; ** *p* < 0.01; *** *p* < 0.001. The scale is 1 cm.

Then, 12 h after pupae emergence and injection with the activator and inhibitor, unfertilized eggs of female moths were dissected and collected. The activity of MPF was detected at different interference concentrations. We observed that MPF activity increased significantly with higher concentrations of the activator and decreased significantly with higher concentrations of the inhibitor (Figure 4A). Simultaneously, we observed ovariole integrity and egg development at varying concentrations. After both activator and inhibitor interference, the eight ovarioles remained fully arranged on both sides of the abdomen, and there was no significant difference in the development status and shape of the eggs compared with the control (Figure 4B). These results indicated that the activator and inhibitor effectively regulated MPF activity without having toxic effects on egg development.

### 3.5. MPF Activity Affected Parthenogenesis Induction

**Figure 5:**
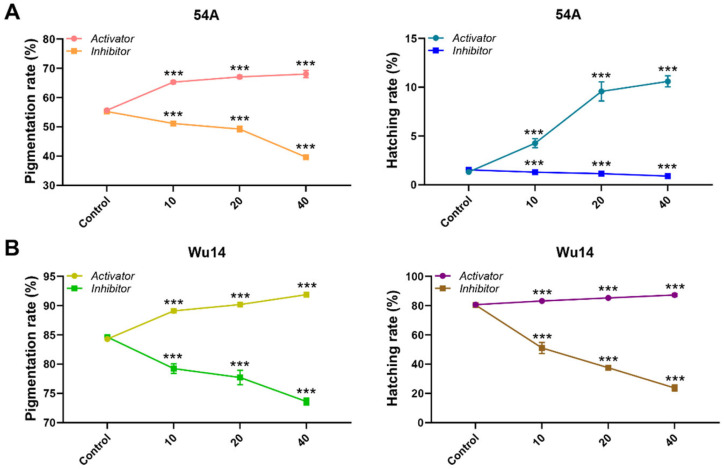
Pigmentation and hatching rates. (**A**) Amphigenetic line 54A. (**B**) Parthenogenetic line Wu14. The unit of concentration was μM. The data shown are means ± S.E.M. (*n* = 3). Asterisks indicate significant differences with a two-tailed *t*-test: *** *p* < 0.001.

We performed parthenogenesis induction on unfertilized eggs after modulating MPF activity and assessed the rates of pigmentation and hatching. Both the parthenogenetic line Wu14 and corresponding amphigenetic line 54A exhibited significant changes in the rates of pigmentation and hatching in response to alterations in MPF activity (Figure 5). Specifically, the upregulation of MPF activity using the activator led to a marked increase in pigmentation and hatching rates for both 54A and Wu14. Conversely, the downregulation of MPF activity resulted in particularly significant reductions in these rates. These results demonstrated MPF’s pivotal role in parthenogenesis initiation, with greater effects observed in the specialized Wu14 line.

### 3.6. Inhibition of MPF Activity Leads to Abnormal Embryonic Development

**Figure 6:**
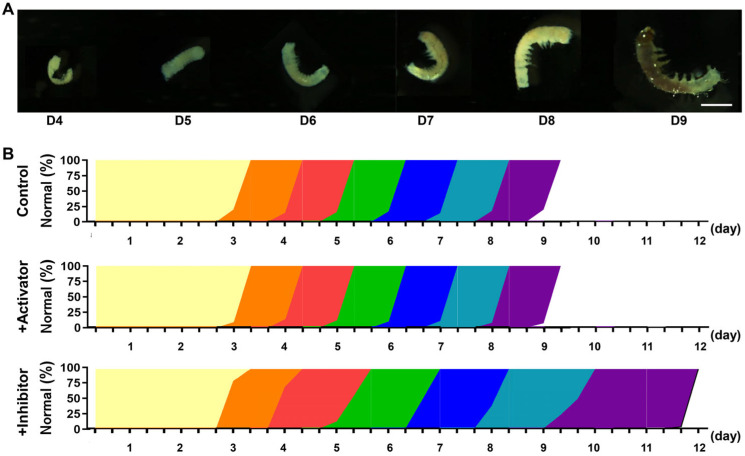
Embryonic development. (**A**) Embryonic morphology. The scale is 1 mm. (**B**) Developmental days. Yellow is D1~D3; orange is D4, the differentiation stage of the tail segment; red is D5, the somatic inversion stage; green is D6, the epidermis formation stage; blue is D7, the tissue formation stage; cyan is D8, the bluish stage; purple is D9, the completion stage of full development.

To systematically evaluate the impact of MPF activity on embryonic development, we conducted detailed morphological analyses of Wu14 embryos under different treatment conditions. The differentiation stage of the tail segment began on the 4th day and reached full development by the 9th day (Figure 6A). On D4 (the differentiation stage of the tail segment), anterior germ segments fused to form head structures; posterior segments developed into a narrow tail region; and a clear distinction between abdominal and tail regions was established. On D5 (the somatic inversion stage), embryonic shortening was observed; ventral curvature in the tail segment was initiated; and the transition from S-shaped to C-shaped morphology took place. On D6 (the epidermis formation stage), complete C-shaped body conformation occurred, and tubercle-like epidermal protrusions emerged. On D7 (the tissue formation stage), the development of body setae (bristle structures) was observed; thoracic hook claws were formed; mandibular serration appeared; and organ pigmentation was initiated. On D8 (the bluish stage), progressive head sclerotization (brown pigmentation) took place, and body pigmentation was initiated but less pronounced. On D9 (the completion stage of full development), full embryonic development was achieved along with preparation for eclosion. Compared to the control, embryos developed synchronously after the activator treatment, while those treated with the inhibitor began to show a developmental delay from day 5 (Figure 6B). This temporal disruption suggests that MPF activity below a critical threshold triggers a progressive developmental delay, ultimately leading to complete developmental arrest in severe cases. These findings unequivocally establish MPF as both a gatekeeper and pacemaker of successful parthenogenetic development, coordinating multiple checkpoints throughout embryogenesis.

## 4. Discussion

Maturation-promoting factor (MPF) serves as a master regulator of oocyte meiosis and cell cycle progression, with its functions well characterized in vertebrate models such as *M. musculus* and *X. laevis* [11,37,38,39]. However, little is known about parthenogenesis in insects, especially in model lepidopteran insect *B. mori*. Catalytic subunit Cdc2 and regulatory subunit CycB cooperate to form the MPF complex [20]. Random coil regions typically exhibit high structural flexibility, which may facilitate the recognition and phosphorylation of these sites by kinases. Phosphorylation is a critical step in regulating the activity of Cdc2, enabling it to bind with CycB and form the active MPF, thereby driving cell cycle progression. This structural arrangement facilitates kinase recognition and subsequent phosphorylation events that regulate Cdc2 activity, enabling its binding with CycB to form functional MPF complexes capable of driving cell cycle progression. The conserved physicochemical properties and structural features of these subunits across species underscore their fundamental role in maintaining precise control over cellular growth, development, and reproductive processes.

After successive generations of breeding and trait accumulation, the pigmentation and hatching rates (≥85% and ≥80%, respectively) of silkworm parthenogenesis induced by warm bath treatment has reached the production application level [40]. The mRNA expression levels of *Cdc2* and *CycB* genes were significantly different between parthenogenetic line Wu14 and corresponding amphigenetic line 54A. In particular, the mRNA expressions of *Cdc2* and *CycB* in Wu14 were significantly downregulated following warm bath induction. Although the mRNA expression levels of Cdc2 and CycB genes significantly decreased after warm bath induction in Wu14, they were higher in Wu14 compared to 54A before induction. This phenomenon may be attributed to the fact that the parthenogenetic line Wu14 has undergone continuous breeding selection for over 30 generations, resulting in the stabilization of reproductive physiological traits. By the unfertilized egg stage, Wu14 may have already accumulated sufficient mRNA expressions of Cdc2 and CycB. This pre-accumulation allows the encoded proteins to undergo phosphorylation upon warm bath induction, enabling Cdc2 and CycB to form the active MPF. In short, the higher expression levels of Cdc2 and CycB genes in Wu14 before induction may reflect an adaptive mechanism to ensure the rapid activation of MPF after warm bath stimulation, thereby promoting cell cycle progression. The activation of MPF depends on the accumulation of CycB to a threshold level, which triggers the transition of Pre-MPF to its active form (Active-MPF), initiating the G2-to-M phase transition and meiosis [18,41]. The result suggested that MPF was closely related to silkworm parthenogenesis.

Beyond initiation, MPF plays equally critical roles in orchestrating embryonic development through the regulation of cell division and differentiation [42]. The positive correlation between MPF activity and the parthenogenesis induction success was verified by using activator and inhibitor. Furthermore, the inhibition of MPF activity resulted in delayed or even arrested embryonic development. Previous studies in vertebrates have shown that increased MPF activity ensures normal embryonic development, while reduced MPF activity leads to developmental delay [43,44,45]. These results strongly suggested that the MPF activity directly affected the process of silkworm parthenogenesis. The enhanced MPF activity may provide the necessary molecular drive to initiate the embryonic development program during parthenogenetic development.

In summary, our study establishes MPF activity as a critical determinant of parthenogenetic success in *B. mori*. Phylogenetic sequence analysis showed that the proteins encoded by the *Cdc2* and *CycB* genes are moderately conserved across various species. These findings not only elucidate the molecular mechanism underlying parthenogenesis induced by warm bath treatment but also enhance our understanding of insect reproductive processes. In addition, our study demonstrates that the MPF is potential molecular target for genetic-based population regulation.

## Figures and Tables

**Table 1:** Specific primers.

Primer Name	Primer Sequence (5′–3′)
BmCdc2-F	ATGGACTCCCTTGGTTCTGG
BmCdc2-R	CACTGGAACACCGAATGCTC
BmCycB-F	ATGTGCAAACTGGGCTTCTG
BmCycB-R	CTGCTAGTTCTGACGGTCGA
BmRP49-F	TCAATCGGATCGCTATGACA
BmRP49-R	ATGACGGGTCTTCTTGTTGG

**Table 2:** The protein sequence numbers.

Species Name	GenBank Accession Numbers		Order
	Cdc2	CycB	
*Bombyx mori*	NP_001037512.1	NP_001037343.1	Lepidoptera
*Manduca sexta*	XP_030037649.1	XP_030029390.1	Lepidoptera
*Trichoplusia ni*	XP_026740648.1	XP_026731829.1	Lepidoptera
*Ostrinia furnacalis*	XP_028177540.1	XP_028156235.1	Lepidoptera
*Amyelois transitella*	XP_013193284.1	XP_013191183.2	Lepidoptera
*Helicoverpa zea*	XP_047033055.1	XP_047023653.1	Lepidoptera
*Battus philenor*	XP_068623488.1	XP_068626558.1	Lepidoptera
*Plodia interpunctella*	XP_053617113.1	XP_053610622.1	Lepidoptera
*Diprion similis*	XP_046738554.1	XP_046746195.1	Hymenoptera
*Neodiprion lecontei*	XP_015520372.1	XP_046598379.1	Hymenoptera
*Athalia rosae*	XP_012254072.1	XP_012254238.2	Hymenoptera
*Hermetia illucens*	XP_037903055.1	XP_037904258.1	Diptera
*Culicoides brevitarsis*	XP_063700522.1	XP_063700924.1	Diptera
*Episyrphus balteatus*	XP_055836824.1	XP_055837226.1	Diptera
*Gryllus bimaculatus*	GLH05238.1	GLG94543.1	Orthoptera
*Anabrus simplex*	XP_067015382.1	XP_066990889.1	Orthoptera
*Schistocerca nitens*	XP_049799429.1	XP_049815951.1	Orthoptera
*Homo sapiens*	NP_001307847.1	NP_114172.1	Primates
*Jaculus jaculus*	XP_004657918.1	XP_045016300.1	Rodentia
*Myxocyprinus asiaticus*	XP_051502898.1	XP_051559981.1	Cypriniformes

## Data Availability

The original contributions presented in this study are included in the article. Further inquiries can be directed to the corresponding authors.
